# Modelling the timing of migration of a partial migrant bird using ringing and observation data: a case study with the Song Thrush in Italy

**DOI:** 10.1186/s40462-023-00407-z

**Published:** 2023-08-01

**Authors:** Roberto Ambrosini, Simona Imperio, Jacopo G. Cecere, Alessandro Andreotti, Lorenzo Serra, Fernando Spina, Niccolò Fattorini, Alessandra Costanzo

**Affiliations:** 1 Dipartimento di Scienze e Politiche Ambientali, Università degli Studi di Milano, Via Celoria 26, Milano, 20133 Italia; 2 Area Avifauna Migratrice, Istituto Superiore per la Protezione e la Ricerca Ambientale (ISPRA), Via Ca’ Fornacetta 9, Ozzano dell’Emilia (BO), 40064 Italia; 3 Present Address: Via della Madonnina, 30 ? I 65010 , Italia, Spoltore (PE), 65010 Italia; 4 Dipartimento di Scienze della Vita, Università degli Studi di Siena, Via P.A. Mattioli 4, Siena, 53100 Italia; 5 NBFC, National Biodiversity Future Center, Palermo, Italia

**Keywords:** Citizen science data, Migration phenology, Ringing data, Short-distance migration, *Turdus philomelos*

## Abstract

**Background:**

The study of the timing of migration is fundamental to the understanding of the ecology of many bird species and their response to climate change, and it has important conservation and management implications e.g., for assessing the hunting seasons according to the EU Directive 2009/147/EC (Birds Directive).

**Methods:**

We developed a new method for the analysis of ringing data (both first capture and re-encounters) and citizen science observations, to assess the timing of pre- and post-nuptial migration of birds. This method was tested on the Song Thrush *Turdus philomelos*, using i) the Bird Ringing Database hosted by the ISPRA Italian Ringing Centre from the whole Italian peninsula, the three closest large islands (Sicily, Sardinia and Corsica), and Canton Ticino (Switzerland) and ii) the eBird data for the same study area.

**Results:**

The results from both datasets consistently showed that pre-nuptial migration starts during the first 10-day period of January (Jan 1) in some central and southern areas of the Italian peninsula, in central Sicily, southern Sardinia, and Corsica. The onset of migration occurs on Jan 2 in the rest of central and southern Italy, Sicily and Sardinia, and western Liguria, while it starts later in the north-eastern Alps, up to Mar 3. The end of post-nuptial migration is more synchronous, occurring on Nov 1 across most of Italy, slightly earlier (Oct 3) in northern Italy and later (Nov 2) in Sicily. The uncertainty of the estimated dates was < 2 days in most cases.

**Conclusion:**

This method represents a novel and valuable tool for the analyses of the timing of migration using ringing and citizen science data and provides an important contribution to the Key Concepts Document of the EU Birds Directive, where migration timings are considered and used to define the hunting period of birds.

**Supplementary Information:**

The online version contains supplementary material available at 10.1186/s40462-023-00407-z.

## Introduction

Migration is a ubiquitous phenomenon in the animal world, described as the seasonal, fine-tuned movements between two areas along a precise route [1, 2]. Every year, billions of animals move across the planet to exploit food resources, find the best breeding grounds and, more generally, minimise exposure to extreme climates. Migration has been described in a large variety of taxa, including insects, fishes and mammals; however, the spectacular journeys undertaken by birds made them the preferred model for migration studies [3]. Migratory birds have a pivotal role in shaping the structure and functionality of ecosystems, linking geographically distant areas and transporting nutrients, pollen, seeds, and organisms such as small invertebrates and microorganisms, including pathogens [4–7].

Albeit widespread, migration does not occur in all bird species, and within a large number of them, not all individuals migrate. Resident species live year-round in the same area, exploiting the locally available resources. Facultative migratory species can migrate or remain in their territories in response to food availability or weather conditions [8]; partial migrants, in which part of a population migrates whereas the other is sedentary throughout the year, are included in this class [9]. Lastly, obligate migrants are species that show a regular, programmed migration, in which individuals move between breeding and non-breeding areas [8]. Migrants can also be separated into “long-distance” migrants, that travel across biogeographic realms (e.g. between the Palearctic and Afrotropical realms), and “short-distance” ones, that travel within the boundaries of one biogeographic realm [8].

Among the studies of migration particular interest has been devoted to the timing (phenology) of movements [10]. Indeed, migration timing has a key role in individual fitness and needs to be finely tuned to environmental conditions at departure areas, along the migration routes, and upon arrival. A technical note on the terminology used in the present work is necessary here: although terms like ‘spring’ and ‘fall’ or ‘autumn’ migration or ‘wintering’ are commonly used in the ornithological literature, they are relative terms as they refer to the timing of seasons in the temperate areas of the boreal hemisphere and therefore do not have a universal meaning. For this reason, here we will use ‘pre-’ and ‘post-nuptial’ migration as these terms have a non-equivocal interpretation directly linked to the stages of the annual life-cycle of a migratory bird.

One year of life in migratory species (particularly obligate and long-distance ones) is characterized by different phases that have to be accomplished in very specific time frames, including pre-nuptial activities such as pre-nuptial moult, partial development of the gonads, migration towards the breeding territories, breeding activities, post-nuptial moult, and migration towards the non-breeding areas [11]. The arrival date in the breeding areas is particularly relevant: earlier arriving individuals can fully exploit the territory resources for breeding activities and, consequently, can have a higher mating success and offspring survival [12], thus increasing their breeding success and therefore their fitness [13–16]. Phenology has also proven relevant to assess the resilience of migrants to climate change [17] because species and populations that advanced their phenology more are also in a better conservation status [18]. However, arrival cannot be advanced too much by individual migrants, as they might be at high risk of incurring spells of adverse weather and/or poor environmental conditions hampering their survival prospects [1, 19]. Similarly, after breeding, birds have to leave the breeding grounds before the arrival of unfavourable environmental conditions and need to reach the non-breeding grounds in time to go through all the stages of their annual life cycle necessary to be ready to migrate again for the next breeding season. Importantly, the post-nuptial migration is remarkably less investigated than the pre-nuptial one, and very few works studied its phenology [20, 21].

A proper assessment of the timing of bird migration is also extremely relevant for bird management and conservation. For instance, art. 7.4 of the European Union (EU) Directive 2009/147/EC (Birds Directive) states that huntable migratory birds must not be harvested “during their return to their rearing grounds” (i.e. pre-nuptial migration). Therefore, EU Member States were requested by the European Commission to indicate the ten-day period (TDP hereafter or ‘decade’ *sensu* the guidance documents of the European Commission on hunting) during which the onset of the pre-nuptial migration of each huntable species occurs within their territories. In 2001, these periods have been included in a formal document (the so-called Key Concepts Document, KCD) that was updated in 2009 and 2014 to integrate data from the newly accessed Member States. The last version of the KCD was published in 2021 after a long revision process that involved all Member States [22]. This country-by-country approach has resulted in discrepancies among Member States even at the same latitudes due, in particular, to the different methodologies applied to distinguish migrating individuals from non-breeding residents in partial and short-distance migratory species, the latter representing the bulk of species listed as huntable under the Birds Directive.

Most of the current knowledge about migration is based on data provided by ringing activity, a research method that dates back to 1899, based on individual marking using metal rings with a unique alphanumeric code closed on the leg. Bird ringing is still among the most widespread methods in ornithology around the globe, being cheap, suitable for all bird species and ideal for long-term and large-scale studies. Unfortunately, it has long been recognized that the sampling effort in the collection of ringing data, as well as the probability of recovery of ringed individuals, are largely heterogeneous over space and time [23, 24]. Therefore, to avoid biases, analyses of ringing data need to take carefully into account their heterogeneity.

Recently, some of the biases related to ringing activity, especially those referred to the inadequate knowledge of the geographical distribution of species (the so-called Wallacean shortfall) [25], have been overcome thanks to the spreading of citizen science, i.e. the collection and analysis of data with the involvement of members of the general public (e.g. amateur ornithologists). Citizen science data have gained importance in recent years thanks to modern technologies (e.g. smartphones and the internet) that allow the collecting, transmitting and storing of large amounts of information [26], potentially even larger than those provided by ringing data. However, the use of citizen science data in the scientific literature is still in its infancy, and methods need to be developed to disclose their full potential [27].

Ringing data have long been used to assess the timing of migration, for instance by analysing the seasonal frequency of encounters [28, 29]. Ring re-encounters of the Barn Swallow *Hirundo rustica* have been the focus of an analysis aimed to propose a new modelling approach to describe the timing of bird migration [30] based on conditional auto-regressive (CAR) models fitted to the cumulated proportion of individuals that are encountered in a given geographical area in different dates. Importantly, this method opened the possibility of using also ringing data (i.e., data about the ringing event, including individuals that were no longer encountered after ringing), − which are much more abundant than ring re-encounters but also potentially even more spatially and temporally heterogeneous − as well as citizen science data (e.g., those stored in the eBird portal; https://ebird.org). These possibilities, however, have not been explored so far using the method of Ambrosini et al. [30].

Another limit of Ambrosini et al. [30] method is that the identification of the date at which a given proportion of individuals reaches a given area is based on the assumption that no individual is present in the study area at the beginning of the investigated season. Indeed, this method was developed to analyse the ring re-encounters in Europe of a long-distant, obligate migrant, such as the Barn Swallow, which spends the non-breeding period in Africa and arrives in Europe in spring. Due to these features, the application of this method could not be extended to the analysis of data of partial migrants (i.e., including both resident and migrating individuals), or of species whose non-breeding staging sites are within the geographical range investigated. This limit is particularly relevant because overcoming it would allow extending the analysis of the timing of migration to a whole migration system and therefore assessing, among other aspects, the departure times from the non-breeding staging sites, a piece of information that is lacking for many species, in particular for those that, in the Eurasian-African migratory systems, migrate to sub-Saharan Africa.

In the present work, we propose and validate a new method for the analysis of the timing of bird migration using ringing and citizen science data that can overcome the limits of the previous work by Ambrosini et al. [30]. To this end, we exploited ringing data of the Song Thrush *Turdus philomelos* included in the Bird Ringing Database at the ISPRA Italian Ringing Centre (www.epe.isprambiente.it) and citizen science data included in the eBird portal [31] to investigate the timing of migration over the Italian peninsula, the three closest large islands (Sicily, Sardinia and Corsica) and Canton Ticino (southern part of Switzerland). In particular, we aimed at assessing the dates at which a proportion of individuals has reached a given locality (for the pre-nuptial migration) or has still to leave it (for the post-nuptial migration). To do so, we adapted the analytical procedure described by Ambrosini et al. [30] on the Barn Swallow to fit data on a short-distance, intra-Palearctic migratory species, improved it in several aspects, and cross-validated this new method by showing that it provides consistent estimates of the onset of migration when applied to the two datasets.

## Methods

### Model species

The Song Thrush is a medium-small size, partially migratory passerine bird of conservation and management interest because it is huntable in Europe. It is a polytypic species belonging to the Turdidae family (Rafinesque, 1815) that includes three subspecies: *T. p. clarkei*, *T. p. hebridensis*, and *T. p. philomelos*, the nominate subspecies, to which Song Thrushes migrating across Italy belong [32]. Populations from northern Europe and the southern Scandinavian Peninsula are obligate migrants, while populations of the southern part of Europe can display either migratory or sedentary behaviour. Italy is a crossroad for individuals breeding in different parts of Europe. Ringing and recovery data are abundant in the Italian Bird Ringing Database (> 150,000 records) and allowed to identify three main directions followed by Song Thrushes reaching Italy after breeding: (1) individuals from central and eastern Europe travel South-West, flying over the Adriatic Sea; (2) birds from the Baltic region migrate South-South-West, across the Alps; (3) individuals from central-western Europe move towards South-South-East, crossing the Alps [33]. During post-nuptial migration, the first migrants are observed in the third TDP of August (Aug 3) and Sep 1, while the peak of migration occurs on Oct 3 and movements end on Nov 2-Nov 3. Once arrived on the Italian mainland, part of the birds overwinter while others continue migrating towards non-breeding grounds along the coast of the Western Mediterranean and North Africa [33, 34]. Non-breeding recoveries in Italy are mainly located southwest of the Apennines, in the northern and central Tyrrhenian sectors, and in Sardinia [33, 34]. Sicily and the southernmost part of the Italian Peninsula are other important non-breeding staging areas, but they are mainly reached by populations not intensively ringed [34], implying a low number of recoveries. Pre-nuptial migration flyways have not been well characterized yet, but we can expect different migratory routes possibly with different phenologies. It is well known, for example, that many Song Thrushes head North from Tunisia to south-eastern France via Sardinia and Corsica [33]. Early movements along this route occur already in the first TDP of January (Jan 1) [35]. However, according to some authors [36–38], in the central and southern parts of mainland Italy, return movements do not start before mid-February. The same TDP has been suggested for France [22] based on visual observations of birds supposed to be actively migrating by volunteer observers [39] and bioacoustical stations, despite recovery analyses suggesting early movements in January in southern France [32, 40, 41].

### Datasets

The Bird Ringing Database at the ISPRA Italian Bird Ringing Centre (www.epe.isprambiente.it) includes data on all birds ringed in Italy, any of their subsequent re-encounters, and all encounters (including ringing) of birds ringed abroad and re-encountered in Italy. Currently, this dataset includes Song Thrush encounters from 1929 to 2011. It is worth mentioning that at least 90% of Song Thrushes recovered in Italy in known circumstances were shot, therefore birds re-encountered more than once are virtually absent in this dataset. We selected data collected in Italy as well as in Corsica (France) and Canton Ticino (the southern part of Switzerland). Although this may introduce some inconsistency in the dataset, as only re-encounters from Corsica and Canton Ticino (i.e. birds ringed in Corsica or Canton Ticino and then recovered in Italy, or birds ringed in Italy and recovered in Corsica or Canton Ticino) are available in the Italian dataset, this approach was justified by the need to encompass birds that were moving along the Sardinia-Corsican route as well as individuals encountered in the southern side of the Alps. Indeed, Song Thrushes from the other parts of Switzerland tend to follow the path of the Rhone River to reach the Mediterranean area of France, from where they move towards the eastern Iberian Peninsula, Balearic Islands and Algeria [32]. Using a conservative approach, the following ringing data were discarded according to previous studies based on ringing encounters [42–44]:

1) birds that were reported as being not freshly dead, birds that were in poor condition or had an accident at ringing, or birds that were alive and probably healthy but taken into captivity (EURING levels for ‘condition’ = 3, 4, 5, 6) [45];

2) birds that were kept longer than 24 h at ringing, or birds that had been moved or held extensively during ringing, or those hand-reared (EURING levels for ‘manipulated’ = C, F, T, M, H);

3) birds that were moved unintentionally by man or other agency, or intentionally by man, or moved by water e.g., found on the shoreline (EURING levels for ‘moved’ = 2, 4, 6);

4) birds for which the dates of ringing and/or recovery were not recorded accurately to the nearest 1 week for both the ringing and the finding date (EURING levels for ‘date accuracy’ = 4, 5, 6, 7, 8);

5) birds for which the places of ringing and/or recovery were not recorded accurately to the nearest 100 km for the ringing or finding places (EURING levels for ‘coordinates accuracy’ = 6, 7, 8, 9).

From the eBird Basic Dataset [46] we retrieved records for the Song Thrush in Italy, Corsica and Canton Ticino from 1900 to 2023. Records from small Italian islands south of 36° N (e.g., Lampedusa and Linosa) were discarded to obtain a geographical extent of data comparable to that of the ISPRA dataset. Indeed, in the ISPRA dataset, data at latitudes < 36° N were too scattered (n = 78) to be included in the analyses. Only reviewed data were retrieved, and no further filtering was applied, as we were interested in the location and date of observations of Song Thrushes, regardless of whether they came from a complete checklist or occasional observations and whether birds were classified as travelling or not. The number of observed individuals was considered whenever the record included this information. Records indicating only the presence of the species were conservatively considered as reports of one individual only. According to these criteria, the eBird dataset included observations of 7474 Song Thrushes.

As we were interested in assessing the timing of the two migration movements by considering the date at which each individual was present in a given location, we used data (either ringing and re-encounter or eBird records) collected between December 1st and May 31st for the pre-nuptial migration, and between September 1st and November 30th for the post-nuptial migration. After applying the above selection criteria, the ISPRA datasets included 13,334 and 113,611 encounters from 1588 to 1945 localities (i.e., different coordinates) for the analysis of pre- and post-nuptial migration, respectively, while the eBird datasets included, respectively, 4437 and 2105 observations from 747 to 296 localities.

### Procedure for the analyses of the timing of the pre-nuptial migration

**Fig. 1:**
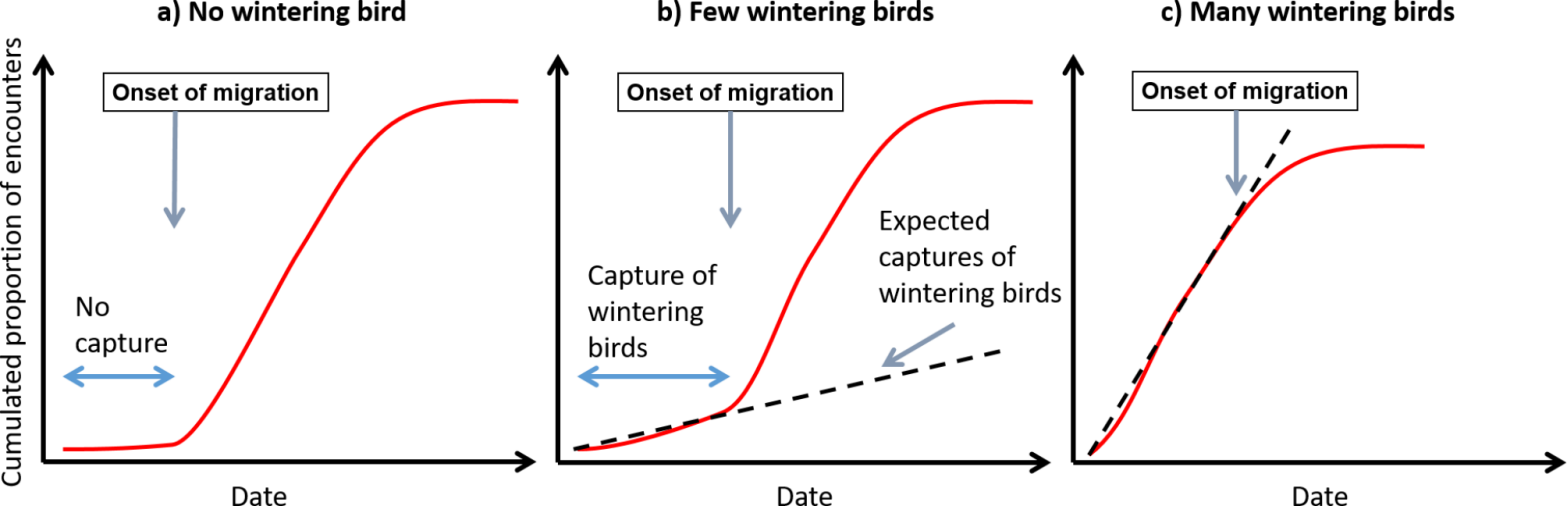
Exemplification curve representing the cumulative proportion of encounters in a cell for **(a)** bird species that do not winter in the study area; **(b)** bird species in cells where few birds winter; **(c)** bird species in cells where many birds winter. In **b**) the onset of the non-stationary period is estimated as the date when the curve deviates from an approximately linear growth in the left tail. In **c**) the onset of migration is estimated by a decrease, rather than an increase, in the proportion of encounters

The analytical approach described in Ambrosini et al. [30] was developed on the ring recoveries data available in the EURING Databank [47] for an obligate, long-distance migratory bird, the Barn Swallow. In the original method, the study area was divided into cells of arbitrary dimension (either 1.5° × 1.5° or 4° × 4° latitude – longitude) and the number of encounters (either ringing data or recoveries) per calendar date per cell was calculated. The number of encounters per day was then transformed first into the proportion of encounters per calendar date and then into the cumulative proportions. The cumulative proportions of encounters at each date and cell were then modelled using a binomial Generalized Linear Mixed Model (GLMM) with a complementary log-log (cloglog) link function (Fig. 1a). An inversion of this model allowed calculating the calendar date when a given cumulated proportion of encounters is expected (Fig. 1a; see Ambrosini et al. [30] for full details on this approach).

**Fig. 2:**
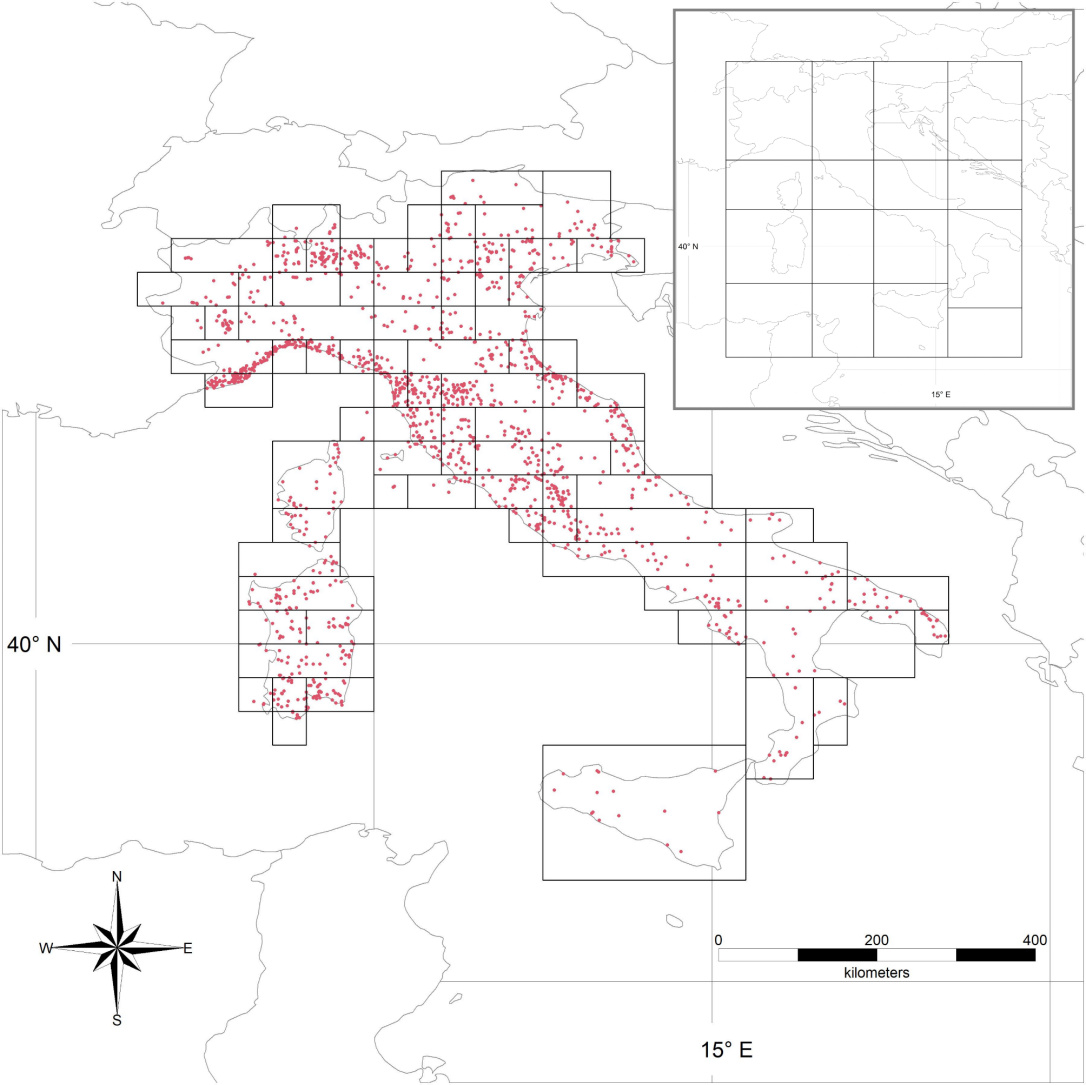
Example of the final result of the cell merging procedure. Red dots are the encounters included in the analysis. Insert shows the areas used for cell creation: only cells within each area could be merged

In the present work, the procedure of Ambrosini et al. [30] has been improved in several ways, including the possibility to fit data on partially migratory species. The main update was the introduction of a procedure for generating cells of different sizes, inversely proportional to encounter density. Preliminarily, a geographical clustering algorithm identifies clusters of encounters that are spatially isolated and are less than the minimum sample size required at a cell. Those data are then discarded. This preliminary step prevents generating unreasonably large cells. The study area (i.e. the minimum bounding rectangle of the remaining encounters) is then divided into cells of 0.5° × 0.5° (latitude - longitude) and the remaining encounters are assigned to a cell. If a cell does not include at least 20 encounters on at least 10 different days of the year (minimum sample size), the cell is merged with the easternmost adjacent cell. If all cells in a 0.5° latitude belt do not reach the minimum sample size even if merged all together, that latitude belt is merged with that immediately north of it. The so-formed belt is then divided longitudinally as above. If the easternmost cell does not reach the minimum sample size, it is merged with the cell west of it. Similarly, if all cells in the northernmost band together do not reach the minimum sample size, the band is merged with that immediately south of it. This procedure, therefore, favours merging cells at the same latitude and joins cells at different latitudes only if the data in a latitudinal band are not sufficient. To avoid merging too many cells in a latitudinal band, this procedure has been applied separately to cells included in different areas (Fig. 2) whose boundaries were designed to include cells with similar ecological features (e.g. for preventing the merging of a cell in Sicily, Sardinia or Corsica with one on the continent). An example of the result of the application of this procedure to ringing data is reported in Fig. 2. The weighted centre (mean of latitude and longitude) of encounters at each cell is then calculated, and its coordinates are used as the coordinates of the cell in the following analyses. This procedure for merging cells thus allows retaining small cells in areas with a high density of data as well as setting large values for the minimum sample size at each cell, which, in turn, allows robust estimates while discarding a few data.

A second main improvement was the use of a GLMM with an exponential spatial covariance structure instead of a CAR model to account for the spatial autocorrelation in the data. This change was necessary because the CAR model necessitates the calculation of a spatial autocovariate, which represents the cumulated proportion of arrivals in the cells neighbouring any given cell. However, the neighbourhood of a cell is irregular both in its shape and in the number of cells considered when cells of different sizes are used, as it happens when cells are merged. For instance, a larger cell probably has more cells in its neighbourhood than a smaller cell. In addition, the calculation of the spatial autocovariate in Ambrosini et al. [30] gives equal relevance to the data in the neighbourhood of a cell, which was reasonable with cells of equal size, but less so with cells of different sizes. Indeed, the inclusion of a large cell in the neighbourhood of a focal cell implies that data potentially far in space from the focal cell are given the same relevance as those from a small cell that is closer in space (for instance, in Fig. 2, the whole Sicily would be in the neighbourhood of the cell in the tip of Calabria). In contrast, the fact that we assigned to each cell the coordinates of the weight centre of the encounters in that cell, allows the spatial GLMM to account for spatial autocorrelation in a way that is more closely related to the real distribution of the encounters. Day-of-the-year (centred on its mean value) was the only fixed predictor in the spatial GLMM, while cell ID was included as a random factor. In addition, the random part included a random intercept and a random slope for the day of the year within a cell (i.e., random intercept and slope model). A formal description of the model is provided in Supplemental Material 1. The GLMM was fitted with the glmmTMB procedure [48] in R 4.0.5 [49], the whole code for the analyses is available in Supplemental Material 2.

A third change was necessary to extend the analyses to species that are present during the non-breeding staging period at least in some parts of the study area. Indeed, for obligate, long-distance migratory species, no encounter is expected during the non-breeding staging period; therefore, the onset of, for instance, pre-nuptial migration can be estimated from model inversion as the date when a given proportion of encounters is expected in a given cell. This criterion, however, does not hold if some individuals are already present in some cells at the beginning of the period investigated, as is the case of partial migrants or species whose non-breeding stationary areas are within the study area. For modelling the migration of those species, it is necessary to assume that in all cells – including those where the birds are stationary – the probability of encountering an individual is larger during the non-stationary as compared to the stationary periods. This assumption is reasonable, as birds have a higher probability of meeting a fixed ‘detector’ (e.g. a ringing station, a hunter) and thus being captured when they start moving for migration than during their stationary periods. The number of encounters is therefore expected to increase during non-stationary periods. In addition, it is necessary to assume that the probability of encountering a non-breeding individual is constant during stationary periods [50, 51]. Under these assumptions, the function interpolating the cumulative proportion of encounters should have a left tail that interpolates the encounters during the stationary period (Fig. 1b). This first part of the curve should grow slowly until the moment when birds enter the non-stationary period, which should correspond to quicker growth of the cumulative proportion of encounters. It is important to note that this growth may be due either to new birds entering the cell or to ‘local’ birds leaving the cell because both processes increase the probability for a bird to be detected. An important assumption of the model is that the first part of the curve can be approximated by a linear function, a deviation from which can be used to assess when the increase in encounters occurs. Another possibility is that the encounter probability is already high at the beginning of the study period, for instance in cells where birds are stationary. In this case, a decline in the encounter probability is observed when the pre-nuptial migration starts. However, also this scenario implies a curve with an approximately linear phase in its left part (Fig. 1c). Thus, under all scenarios, the day of the onset of the non-stationary period at a given cell can be estimated as follows. The cumulative proportion of encounters should be calculated starting from a date when birds are reasonably stationary in all their range. From the cloglog curve fitted by the GLMM, the expected proportion of encounters on each day of the year and cell is estimated. These expected proportions at each cell are then regressed on the date. This latter analysis is initially limited to a period of 10 days starting from the earliest date considered (i.e. in a period when birds are stationary), and the sum of the absolute residuals of the regression is noted. This analysis is then repeated considering a time span of 11 days and so on until the sum of the absolute values of the residuals reaches a threshold arbitrarily set to 0.05. This criterion proved to be more robust than an alternative one based on R-squared values of the regressions, particularly when the fitted curve is already steep at the beginning of the considered period (third scenario above). This approach allows identifying the end of the stationary period of birds and the onset of migration at a cell as well as the day when the curve estimating the cumulated proportion of birds reaches a given value (e.g., 5%) above the cumulated proportion of encounters at the end of the stationary period. Technically, if at the end of the time span the cloglog curve has value $A$, and we want to calculate the day when a proportion $p$ of migrants has arrived on a cell, the procedure calculates the date when the cloglog curve reaches the value $A+p (1-A)$.

Predicted values from the model are then spatially interpolated using a grid with 0.5 × 0.5-degree cells. Interpolation is based on the inverse distance weighting algorithm using the Shepard method to calculate weights [52]. A leave-one-out validation is applied to measure the error in the interpolated values and to choose the best power function for interpolation. Finally, the resulting interpolated map is downscaled to obtain the expected values at cells of 0.1 degrees of size (latitude × longitude) using the bilinear method [53, 54].

The whole procedure includes a set of parameters, listed in Table 1, whose values were chosen arbitrarily. To assess the sensitivity of the method, we re-run the analyses with different values of the parameters. These changes can slightly reduce the overall sample size on which the analyses are based, for instance, because a different start date is selected. By combining the different values of three parameters (i.e. StartD, NP and THR in Table 1), we obtained 27 final maps, one for each value of the predicted proportion of arrived migrants (i.e. for each value of PRED in Table 1). To summarize this information, we calculated a map of the average value at each cell across the 27 downscaled maps for each value of the predicted proportion of arrived migrants. In addition, to estimate the uncertainty of the estimates under different arbitrary choices of the set of parameter values, we calculated a sensitivity map by applying to each cell the formula.

**Table 1:** Parameters entered in the procedure and values used for predictions of the timing of pre-nuptial migration

Name in the procedure	Description	Values used
StartD	**Pre-nuptial migration**: first day-of-the year for calculating cumulated ring encounters **Post-nuptial migration**: last day-of-the year for backward calculation of cumulated ring encounters.	**Pre-nuptial migration** 1 December 6 December 11 December **Post-nuptial migration** 20 November 25 November 30 November
NP	Minimum number of encounters in a cell	15, 20, 25
ND	Minimum number of different day-of-the-year for the encounters in a cell	Values are univocally defined according to NP values: NP = 15 → ND = 7 NP = 20 → ND = 10 NP = 25 → ND = 15
NumDayLin	Minimum length of the linear phase	10
THR	Threshold value of the sum of absolute residuals of the linear regression of the left-hand part of the interpolated curve on time used to assess the end of the linear phase	0.05, 0.07, 0.1
PRED	Predicted proportion of individuals that have arrived used for model inversion	0, 0.01, 0.05, 0.1, 0.5

$$Sensitivity=\frac{1}{26}\sum _{i=1}^{27}{\left({d}_{i}-\stackrel{-}{d}\right)}^{2}$$

where ${d}_{i}$ is the date estimated at that cell from the model with the *i*-th combination of parameter values and $\stackrel{-}{d}$ their mean value i.e.

$$\stackrel{-}{d}= \frac{1}{27}\sum _{i=1}^{27}{d}_{i}$$

Each map, associated with the relevant dataset, can be found at the following link: http://doi.org/10.17616/R31NJMIQ.

### Procedure for the analyses of the timing of the post-nuptial migration

The analysis of the timing of the post-nuptial migration follows strictly the procedure used to model prenuptial migration. The main difference is that, for post-nuptial migration, calendar dates are calculated backwards starting from a period when individuals are stationary (i.e. November 30th, see above). Consistently, cumulated proportions are calculated starting from when birds are stationary in their non-breeding periods and going backwards in time towards the breeding season. The linear phase thus represents the cumulated proportion of encounters of individuals that are stationary in the cell at the end of post-nuptial migration. Similarly to pre-nuptial migration, the interpolated curve should deviate from the linear phase when the proportion of encountered individuals is $p$ above that expected at the end of the linear phase (see above). For post-nuptial migration, the values returned by the procedure thus represent the dates *after which*, according to the model, a proportion $p$ of individuals is encountered. This means that if, for instance, in a cell, the value $p = 0.05$ is estimated to occur on November 1st, 95% of individuals have already been encountered in that cell on October 31st and only 5% is expected to be encountered from November 1st onwards. Thus, for post-nuptial migrations, the maps we produced indicate the end-tail of migration.

### Data rarefaction

The main difficulty in the analysis of ringing data is the large heterogeneity in the encounter probability in space and time. To assess whether this problem may have hampered our results, we developed a procedure for rarefying the data to achieve a more even spatial distribution and applied it to the ISPRA dataset.

In the procedure of analysis described above, cells are created to include a minimum number of encounters on a minimum number of different calendar dates (see above). Data rarefaction occurred after cell creation by randomly removing encounters from a cell until it included only the minimum number of encounters or the minimum number of different days of the year necessary to retain a cell in the analysis. Since both these parameters varied as reported in Table 1, the procedure was repeated for each combination of the parameters, leading to a decrease in the number of encounters used for the analysis of pre-nuptial migration from 12,651 − 13,334 (depending on the values of the other parameters) to 1,537-2,203 and that of post-nuptial migration from 112,530 − 113,611 to 1,463-2,527.

We note that this procedure also accounted, at least partly, for the heterogeneity of ring encounters between seasons, as the sample sizes of the analyses of pre- and post-nuptial migration after rarefaction were almost similar, despite those of the original datasets largely differed. Although this procedure did not account for the heterogeneity of encounter probabilities among years, this problem should be minimized by the fact that we did not consider the year of encounter in any step of the analysis. We acknowledge that an analysis of the phenological shift of Song Thrush migration could be of great interest, but it is beyond the scope of the present work.

## Results

### Timing of pre-nuptial migration

**Fig. 3:**
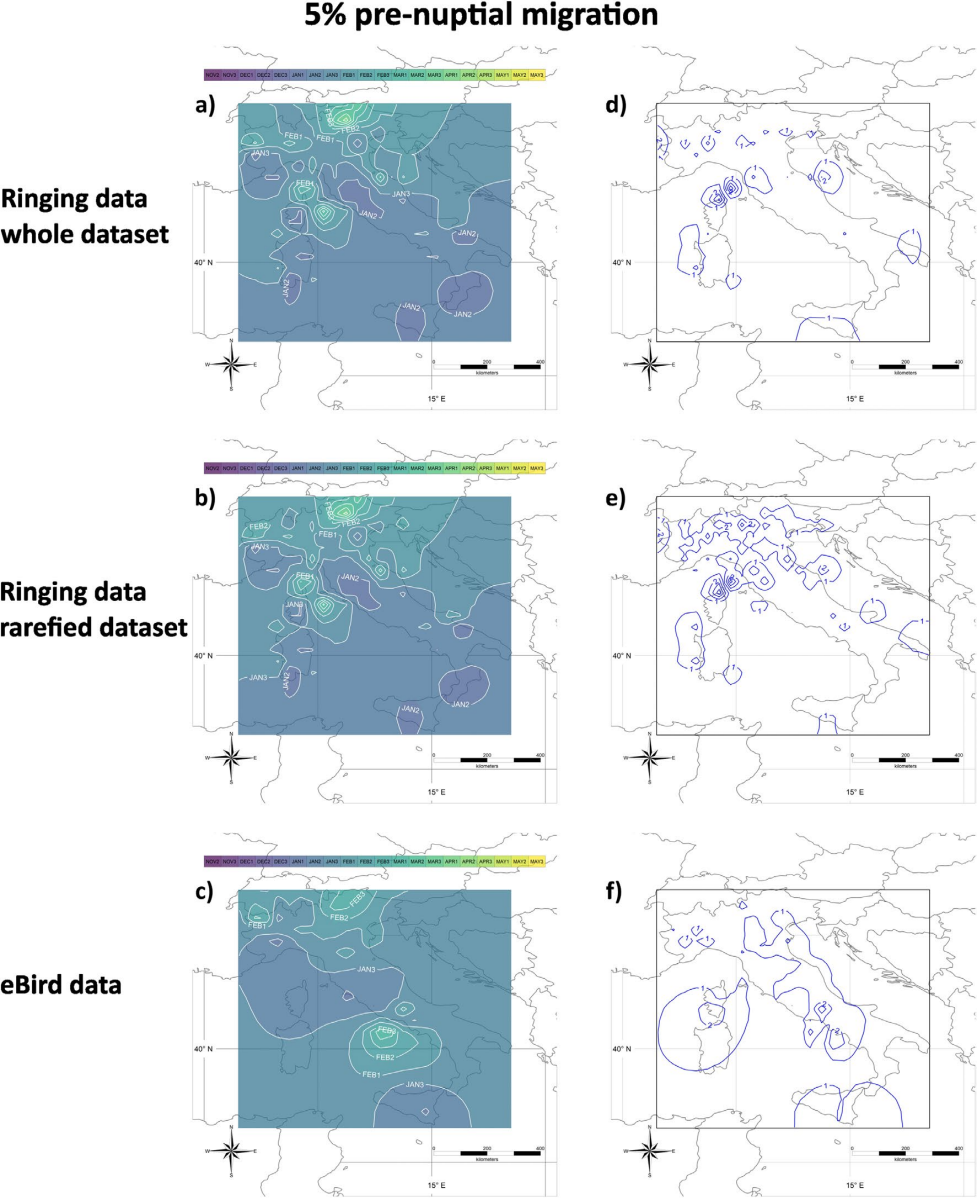
**a**), **b**), and **c**) Onset of the pre-nuptial migration. Dates indicate when 5% more encounters than expected from the capture/observation of stationary individuals do occur; they have been calculated on the whole **(a)** or the rarefied **(b)** ringing dataset, or the eBird dataset **(c)**. Isolines represent areas where the migration date occurs at the same time (e.g., in the central part of Sicily the onset of pre-nuptial migration occurs during the first ten days of January, while in the western and eastern parts of Sicily, it occurs during the second ten days of the same month). Months are divided into ten-day periods (‘decades’ *sensu* the Key Concepts Document of the EU Birds Directive; e.g. Jan 1, Jan 2, Jan 3). Isolines labels should be interpreted as the first day of the corresponding decade, e.g. isoline JAN1 should be read as “01 January”, JAN2 as “11 January”, JAN3 as “21 January” and so on. **d**), **e**) and **f**) Sensitivity analyses associated with panels **a**), **b**), and **c**), respectively. Isolines incorporate areas with the same sensitivity values (in days) of the estimated date of onset of the pre-nuptial migration

**Fig. 4:**
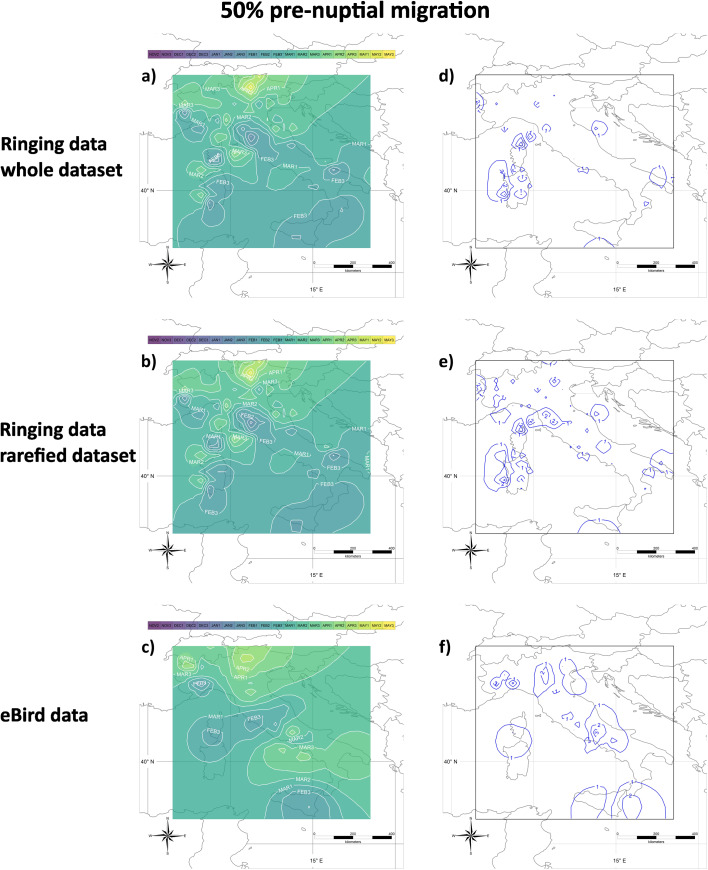
**a**), **b**) and **c**) Median dates of the pre-nuptial migration. Dates indicate when 50% more encounters than expected from the capture/observation of stationary individuals do occur; they have been calculated on the whole **(a)** or the rarefied **(b)** ringing dataset, or the eBird dataset **(c)**. Isolines represent areas where the migration date occurs at the same time (e.g. in **(a)** in the central-southern part of Sicily the median date of pre-nuptial migration occurs during the first ten-day period of February, while in the rest of the island, it occurs mainly during the second ten days of the same month). Months are divided into ten-day periods (‘decades’ *sensu* the Key Concepts Document of the EU Birds Directive; e.g. Jan 1, Jan 2, Jan 3). Isolines labels should be interpreted as the first day of the corresponding decade, e.g. isoline JAN1 should be read as “01 January”, JAN2 as “11 January”, JAN3 as “21 January” and so on. **d**), **e**) and **f**) Sensitivity analyses associated with panels **a**), **b**) and **c**), respectively. Isolines include areas with the same sensitivity values (in days) of the estimated median date of the pre-nuptial migration

The timing of pre-nuptial migration is presented in Figs. 3 and 4, representing the calendar dates when 5% and 50% respectively of encounters of migratory individuals are expected to occur during pre-nuptial migration. More precisely, they are the maps of the mean values of 27 interpolated maps, each obtained by a different combination of parameters, and represent the date when 5% or 50% more encounters than those predicted by the capture of stationary individuals are expected to occur in a cell. Maps relative to different proportions of encounters (0%, 1% and 10%) are available in Supplemental Material 3.

We conservatively set the ‘onset’ of migration at the date when 5% of encounters of migratory individuals occurs. From the analysis of ringing data, the onset of pre-nuptial migration took place during the first TDP of January (Jan 1) in the southern parts of the Italian peninsula, central Sicily, southern Sardinia and Corsica, as well as in an area of central Italy between Tuscany and Lazio, and small areas in the north-west of Italy (Piedmont and Liguria). The onset of pre-nuptial migration occurs on Jan 2 in the rest of central and southern Italy, Sicily and Sardinia, as well as in western Liguria. It occurs on Jan 3 in an area from eastern Liguria to Canton Ticino and on Feb 1 along the north Adriatic coast. Some areas in the Alps see a later onset, up to Mar 3 (Fig. 3a). These results remained substantially unaltered when the data were rarefied, displaying only a ten-day delay in the arrival in Canton Ticino, reached by Feb 1 (Fig. 3b). Results of the analyses on eBird data identified a timing of onset of migration similar to that from ringing data. Indeed, maps showed an onset on Jan 1 in Sicily, in central Italy between Tuscany and Lazio, and the northwest of Italy. It is between Jan 3 and Feb 1 in most of Italy, with a later onset, up to Feb 3 in the Alps and in the small islands of the Tyrrhenian Sea (Ponza, Ventotene and Capri; Fig. 3c).

The sensitivity of the estimates obtained by slightly changing the parameters on which the model is based is less than one day in most of Italy, and reaches a maximum of four days in the maps on ringing data close to northern Corsica, where data density is lower because only recoveries of birds ringed in Italy are available (Fig. 3d). Sensitivity is slightly larger when rarefied data are used, as expected due to the much lower sample size, with larger areas with values between 1 and 2 days (Fig. 3e). It is less than 2 days in most of Italy in the analyses of eBird data (Fig. 3f).

The median number of encounters of individuals in pre-nuptial migration (median date of migration) from ringing data occurs on Feb 1 in small areas of Sicily, Sardinia, Apulia, Corsica and Tuscany and on Feb 2 in larger areas surrounding them and in western Liguria. It occurs on Feb 3 in the central-southern part of Italy, and on Mar 2 in northern Italy and Canton Ticino. Lastly, the median date of migration in the eastern part of northern Italy occurs in April (Fig. 4a). These results remained qualitatively unchanged when data were rarefied (Fig. 4b). Results on eBird data showed a later median migration, on Feb 2 in Sicily, central Italy, and between Sardinia and Corsica. It is on Mar 2 or Mar 3 in southern Italy and in the northwest of Italy, and later, up to Apr 3, in the northeast and the Alps (Fig. 4c).

The sensitivity of estimates on ringing data is generally < 1 day in most of the study area, with a maximum value of 3 days in small areas of Sardinia and Corsica (Fig. 4d), with slightly larger values, but always < 4 days, on both rarefied data (Fig. 4e) and eBird data (Fig. 4f).

### Timing of post-nuptial migration

**Fig. 5:**
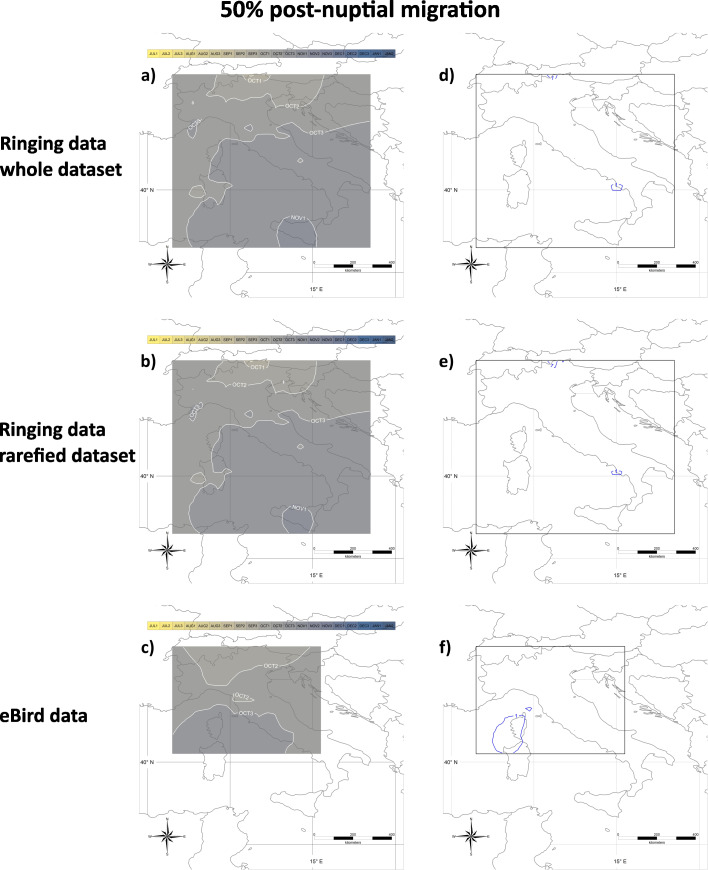
**a**), **b**) and **c**) Median dates of the post-nuptial migration. Dates indicate when 50% more encounters than expected from the capture/observation of stationary individuals have still to occur; they have been calculated on the whole **(a)** or the rarefied **(b)** ringing dataset, or the eBird dataset **(c)**. Isolines represent areas where the mean migration date occurs at the same time (e.g., in **(a)** in the central part of Sicily the median date of post-nuptial migration occurs during the first ten-day period of November). Months are divided into ten-day periods (‘decades’ sensu the Key Concepts Document of the EU Birds Directive; e.g. Oct 1, Oct 2, Oct 3). Isolines labels should be interpreted as the first day of the corresponding decade, e.g. isoline OCT1 should be read as “01 October”, OCT2 as “11 October”, OCT3 as “21 October” and so on. **d**), **e**) and **f**) Sensitivity analyses associated with panels **a**), **b**) and **c**), respectively. Isolines include areas with the same sensitivity values (in days) of the estimated median date of the post-nuptial migration

**Fig. 6:**
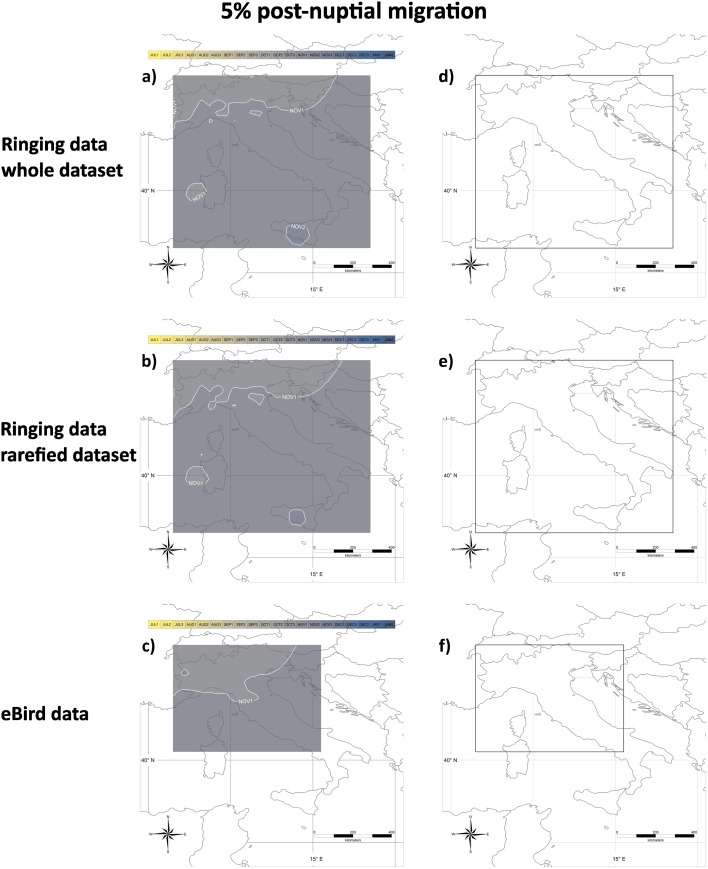
**a**), **b**) and **c**) End of the post-nuptial migration. Dates indicate when 5% more encounters than expected from the capture/observation of stationary individuals have still to occur; they have been calculated on the whole **(a)** or the rarefied **(b)** ringing dataset, or the eBird dataset **(c)**. Isolines represent areas where the mean migration date occurs at the same time (e.g., in **(a)** in the central part of Sicily the end of the post-nuptial migration occurs during the second ten days of November). Months are divided into ten-day periods (‘decades’ sensu the Key Concepts Document of the EU Birds Directive; e.g. Nov 1, Nov 2). Isolines labels should be interpreted as the first day of the corresponding decade, e.g. isoline NOV1 should be read as “01 November”, NOV2 as “11 November” and so on. **d**), **e**) and **f**) Sensitivity analyses associated with panels **a**), **b**) and **c**), respectively. Isolines include areas with the same sensitivity values (in days) of the estimated date of the end of post-nuptial migration

The results of the analysis of post-nuptial migration are represented in Figs. 5 and 6 showing the dates when a given proportion of encounters of migratory individuals has still to occur. As shown in Fig. 5a, the median post-nuptial migration date in Italy shows a gradient from Sep 3 in north-eastern Italy (Sudtirol, in Italian Alps), to Nov 1 in Sicily. In north-western Italy, the median migration date is Oct 2. Results did not change substantially in the analysis of rarefied data (Fig. 5b). Results on eBird data showed a later median migration date on Oct 1 in north-eastern Italy, and are limited to central Italy because data were too scattered in southern Italy. Sicily, and Sardinia (Fig. 5c). The sensitivity was < 2 days for all the analyses (Fig. 5d and f).

The TDP after which only 5% more encounters than those expected by stationary individuals occur, which we consider the date of the end of post-nuptial migration, is Nov 1 across most of Italy, slightly earlier (Oct 3) in northern Italy and later (Nov 2) in Sicily (Fig. 6a). Also in this case, results did not change substantially when we repeated the analyses on rarefied data (Fig. 6b) or eBird data (Fig. 6c). Sensitivity was < 2 days in all the analyses (Fig. 6d and f).

Maps relative to different proportions of encounters (0%, 1% and 10%) are available in Supplemental Material 4.

## Discussion

In this paper, we modelled the timing of migration of a partial migrant, the Song Thrush, based on ringing data available in the databank at the ISPRA Italian Ringing Centre (www.isprambiente.gov.it) and on data from the eBird (ebird.org) citizen science dataset. To this end, we built on a previous method developed by Ambrosini et al. [30] to model the migration progression of a long-distance migrant, the Barn Swallow, which was improved to allow modelling species that are present during the whole year at least in part of the study area. We also cross-validated the results between the two datasets and against the most well-known weakness of ringing data, i.e. the large spatial heterogeneity in the sampling effort. Below, we discuss the main novelties of the method proposed here, the results obtained and their implication for the conservation and management of the Song Thrush.

### Main advantages of the new analytical framework

The method of analysis applied here extends that proposed by Ambrosini et al. [30] in several ways. First, it is designed to apply not only to species that are absent from the area of investigation for a part of the year (e.g. during the boreal winter) but also to species that are present year-round in part of the area. This does not prevent the method to be applicable also to fully migratory species and makes it a generalization of the previous one.

The present method assumes that the probability that individuals present in an area (e.g., because they are overwintering there) are recovered is low and constant during their stationary period, and increases when migration movements start. This is biologically reasonable, as an increase in the individual movements should translate into a larger fraction of them that are encountered (i.e., either captured, re-captured or observed). The new method also introduces a procedure for a flexible definition of cells, whose size increases in areas where data density is lower. This has multiple benefits. First, it allows using small cells in data-rich areas, thus increasing the precision of the results. Second, it allows using the vast majority of the available data. Third, it allows setting a minimum number of data available per cell that guarantees robust model performances (see below). Indeed, in areas where data are more sparse, cells are enlarged until these minimum requirements are met. The method also rests on a set of parameters whose values were arbitrarily chosen (see Methods and Table 1). To reduce the potential effect of such arbitrary choices on the final results, we combined in a final average map the results obtained by combing slightly different values of these parameters in 27 different ways. Importantly, such results were largely consistent, independently of the choice of the parameter values, as their sensitivity was < 2 days in most of the study area, and always < 5 days (Figs. 3, 4, 5 and 6 and Supplemental Materials 3–4).

Ringing data are known to be affected by heterogeneity in their distribution in space and time. The impact of variability between years should be reduced with this method of analysis, as the information on the year of encounter is not considered, and the analysis rests only on the day of the year when an encounter occurred. We investigated the robustness of the method to the large spatial heterogeneity of ring encounters through a data rarefaction procedure that reduced the number of encounters at each cell to the minimum number set *a priori* for the analysis. Overall, this rarefaction reduced the number of encounters used by ~ 10 times for the analyses of pre-nuptial migration and by ~ 100 times for those of the post-nuptial one. Despite such a large reduction in the number of data, the final maps are very similar, with only a small increase in the sensitivity of the results based on rarefied data (Figs. 3, 4, 5 and 6 and Supplemental Materials 3–4). Such consistency in the results, on the one side, demonstrates the robustness of this analytical approach; on the other side, it is not surprising, because the basic procedure of the method rests on the interpolation of a curve at each cell, and we set minimum values for the number of data available per cell that allow a robust interpolation. Thus, this robustness is due to the combination of a robust minimum value for sample size and the availability of a flexible procedure for cell identification that guarantees to reach the threshold at each cell without losing much data.

The only condition that may introduce local biases both in the analyses of all ringing data and in those on rarefied ones is when an intense ringing activity is systematically carried out in a locality (e.g. a ringing station) only during part of the migration period, and these data represent all or most data in a cell. The impact of these possible biases on final results should be reduced by running the analyses with one global model that accounts for the spatial autocorrelation of the data and thus reduces the differences among nearby cells. In addition, the main limit of observational data from citizen science projects is that the identity of an individual is not recorded, so the same bird can be reported by multiple birders on the same day. How and how much this can bias the results is still to be assessed. However, the consistency in the results obtained from the two datasets, at least in the identification of the earliest ten-day period of onset of migration, which is the most important result for applicative purposes, further confirms the robustness of the results of the present elaborations.

### Timing of Song Thrush migration

Our analytical method identified the onset of pre-nuptial migration of the Song Thrush in Italy on Jan 1, consistent with the period reported in the Key Concepts Document [22]. The earliest movements occur in Sicily, central Italy and, according to ringing data, also in southern Sardinia; early movements in Sardinia and Sicily are consistent with the arrival of individuals from North Africa. The onset of migration is progressively later northwards, with a pattern that is consistent with the northward movements of migrating individuals. Interestingly, the onset of pre-nuptial migration occurs earlier in western than in eastern Liguria, suggesting westward movements of individuals from Corsica towards the continent, which are consistent with previous analyses of re-encounter data only [33] and data from hunting bags [35].

Other studies, based on trends in captures and variations in body conditions (body mass and subcutaneous fat deposits), suggested a later onset of migration for individuals in central-southern Italy, which is closer to the timing of the median migration (50% maps) estimated by our models [36–38]. However, these results are based on relatively few individuals (18–180 individuals per year) captured from Jan 2 which, therefore, could not be representative of the onset of Song Thrush migration and are more likely representing “average” individuals than early ones. We thus consider these results as confirmatory of the median timing of pre-nuptial migration estimated by our models. Similarly, the increase in body mass and fat scores of captured individuals observed in February and March in different areas of central and southern Italy (Latium, Campania and Calabria) [36–38] can be easily reconciled with the present results, as it is possible that in January and early February, these studies captured mostly stationary individuals given the small sample size analysed in these papers. Furthermore, it may be that fatter birds caught later in the season belong to populations heading to more distant (and northern) breeding grounds, while early departing ones may be individuals that move shorter distances and thus do not necessitate building up large fat reserves [55].

Since the method of analysis presented in the present study does not account for the movement of individuals, it may be argued that the detected increase in the proportion of encounters in the pre-nuptial analysis may represent the arrival of individuals in the non-breeding ground after post-nuptial migration. This is unlikely because such an interpretation would imply a *later* estimated timing at more southern latitudes, while the general patterns depicted in Figs. 3 and 4 show the opposite and are consistent with northward rather than southward movements. Another possible criticism is that early movements recorded in January could be the results of cold spells forcing birds to move from their non-breeding grounds in search of more suitable places. However, any cold-induced movement should occur more likely at higher latitudes and produce later estimates of the migration phenology in southern directions, but this is not the case.

A pattern suggesting southward movements is visible in the maps of the timing of post-nuptial migration, where southern areas show a later end of migratory movements, with a very end on Nov 2 (Fig. S3.1), consistent with the migration phenology of the Song Thrush reported in Cramp (1988) [56] (among others). The present method of analysis seems thus able to capture the actual migration patterns of this species both in the pre- and the post-nuptial migration.

### Conservation implications

Migration movements of a bird species through a specific country can be characterised by many routes, used by different populations with possibly different timings. Assessing the migration phenology only on a limited number of sites (and extending the outcome to the whole country) can thus be misleading, especially when the identification of the earliest movements is required for management purposes (as in the case of the Key Concepts Document). Correct identification of the onset of the pre-nuptial migration is crucial to ensure full compliance with art. 7.4 of the EU Birds Directive and to guarantee the protection of an important component of bird populations during a critical and high-energy demanding period of the life cycle, such as migration. Indeed, early migrants are mostly adult males that reach their nesting grounds first, occupy the best territories and produce a higher number of offspring [13, 15, 57]. Usually, they also belong to those sub-populations nesting at lower latitudes, where the optimal conditions for breeding are met earlier [58, 59]. This implies that an extended hunting season does not exert uniform pressure on the whole population of a given game species, having a higher impact on some sub-populations and on population segments likely to have a key demographic role.

### Conclusions and future perspectives

Thanks to our innovative analysis of ring encounters and citizen science data, we provided a detailed model of the timing of the pre- and post-nuptial migration of the Song Thrush across the Italian peninsula and the main Mediterranean islands, with important management and conservation implications for the species. This method can also be easily applied to a diverse set of species [60]. Importantly, the information necessary for this method is simply the coordinates and the date when an individual was observed. Here, this extension allowed applying this method to a dataset which includes all first-encounter data (i.e. data of individuals that were encountered only once), which are more abundant than ring re-encounters and records of unmarked individuals available from citizen science data. This opens the possibility of using an enormous amount of information over large geographical areas and discloses the possibility to define management strategies at a continental scale and with a proper flyway approach. This method may also allow the investigation of the phenological changes in bird migration in response to climate change. Indeed, as in Ambrosini et al. [30], this can be done simply by comparing the results of data collected in the past and more recent years. However, despite its wide interest, this investigation is beyond the scope of the present study.

In summary, this method may represent a robust and valuable tool for the rapid identification of the onset of pre-nuptial migration and the end of post-nuptial one, widely applicable to different datasets and multiple species, and may thus provide much-needed information for conservation and management purposes.

## Electronic supplementary material

Below is the link to the electronic supplementary material.

Supplementary Material 1

Supplementary Material 2

Supplementary Material 3

Supplementary Material 4

## Data Availability

Data and code are currently uploaded as Supplemental material 2 and are available at the following link: http://doi.org/10.17616/R31NJMIQ. eBird data can be downloaded at https://ebird.org/data/download.
