# Supplementary material for: Modelling the timing of migration of a partial migrant bird using ringing and observation data: a case study with the Song Thrush in Italy
Source: Mov Ecol. 2023 Aug 1;11:47. doi: 10.1186/s40462-023-00407-z (PMC10391980; doi:10.1186/s40462-023-00407-z)
Supplement: Supplementary file 1 — Supplementary Material 1 [file 40462_2023_407_MOESM1_ESM.docx]

Spatial binomial generalized linear mixed model

The analytical procedure for interpolating the cumulated proportion of ring encounters or observations (simply ‘encounters’ hereafter) recorded in a cell at each date over the periods of pre- or post-nuptial migration, by also accounting for the spatial autocorrelation of data can be described as follows. Let $n_{jt}$ be the cumulated number of encounters in cell $j$ until date $t$, irrespective of the year of recovery, $N_{j}= \sum_{t=1}^{T} n_{jt}$ be the total number of encounters in cell $j$ and $p_{jt}=n_{jt}/N_{j}$ the proportion of encounters recovered until date $t$, $t = 1, \ldots, T$, $T$ being the end of the period of interest. A binomial regression model can estimate the cumulative proportion of arrivals in cell $j$ at any given date as a function of a set of secondary variables. We modelled the occurrences in a cell as a linear function of the date on a complementary log-log (cloglog) scale since this scale is the most appropriate to model spatial point patterns on a geographical grid [21]. To account for spatial autocorrelation and avoid biased estimates, we specified an exponential autoregressive correlation function for arrivals i.e. a spatial autocorrelation function whereby the intensity of the spatial autocorrelation decreased exponentially with geographical distance. Each cell was assigned coordinates corresponding to the mean latitude and longitude of all the encounters in that cell. In this way, the coordinates of each cell represented the “weight centre” of all the encounters in that cell. To account for the inter-cell variability in the patterns of migration through time, cell identity was entered as a random grouping factor and date as a random slope at the cell level.

More formally the model is specified by

$$\log\left( -\log\left( 1-E\left( p_{jt} \right) \right) \right)=\alpha+\beta t+A_{j}+G_{j}t$$

where $G_{j}\sim N\left( 0,\upsilon^{2} \right)$ and $(A_{1},A_{2},\ldots)\sim\mathrm{MN}\left( 0, \Sigma\right)$, with $N$ the Normal distribution and $\mathrm{MN}$ the multivariate Normal distribution, whose covariance matrix among random effects $\Sigma$ is Power exponential, i.e.

$$\Sigma_{i,j}= Cov\left( A_{i}, A_{j} \right)=\sigma^{2}e^{\left( -\frac{h_{i,j}}{2\phi^{2}} \right)}$$

with $h_{i,j}$ the distance between the weight centres of cells $i$ and $j$ and $\phi$ the scale parameter.

Note that this model accounts for different numbers of observations at each cell as the dependent variable $p_{jt}$ is specified in the model as the ratio between the cumulative number of individuals that have reached cell $j$ until time $t$ over the total number of individuals in the cell (actually $\frac{n_{jt}}{N_{j} - n_{jt}}$ in the procedure that we used for the analysis [25]). The variance of the dependent variable is therefore calculated by taking into account the total number of observations at a cell, thus giving larger weights to cells with more observations.

These analyses were performed by the *glmmTMB* procedure in the *glmmTMB* package [25] in R 4.0.5 [28]. The cloglog function was interpolated by specifying the cloglog link function in the *glmmTMB* procedure. Supplemental Material 2 reports the code for the whole procedure, including that for fitting this model.
