# Supplementary material for: Modelling the timing of migration of a partial migrant bird using ringing and observation data: a case study with the Song Thrush in Italy
Source: Mov Ecol. 2023 Aug 1;11:47. doi: 10.1186/s40462-023-00407-z (PMC10391980; doi:10.1186/s40462-023-00407-z)
Supplement: Supplementary file 2 — Supplementary Material 2 [file 40462_2023_407_MOESM2_ESM.zip › Supplemental material 2/cntry00.htm]

World Countries 2000 (cntry00.shp)


### World Countries 2000 (cntry00.shp)

Metadata also available as

#### Frequently-anticipated questions:

- What does this data set describe?
  1. How should this data set be cited?- What geographic area does the data set cover?- What does it look like?- Does the data set describe conditions during a particular time period?- What is the general form of this data set?- How does the data set represent geographic features?- How does the data set describe geographic features?- Who produced the data set?
    1. Who are the originators of the data set?- Who also contributed to the data set?- To whom should users address questions about the data?- Why was the data set created?- How was the data set created?
        1. From what previous works were the data drawn?- How were the data generated, processed, and modified?- How reliable are the data; what problems remain in the data set?
          1. How well have the observations been checked?- How accurate are the geographic locations?- How accurate are the heights or depths?- Where are the gaps in the data? What is missing?- How consistent are the relationships among the data, including topology?- How can someone get a copy of the data set?
            1. Are there legal restrictions on access or use of the data?- Who distributes the data?- What's the catalog number I need to order this data set?- What legal disclaimers am I supposed to read?- How can I download or order the data?- Who wrote the metadata?

---


### What does this data set describe?

*Title:* World Countries 2000 (cntry00.shp)

*Abstract:*: Boundaries for the countries of the world, as they existed in 2000.

1. **How should this data set be cited?**

   > ESRI, 20001101, World Countries 2000 (cntry00.shp):, ESRI, Redlands, California, USA.
   >
   > Online Links:
   > - none

   - **What geographic area does the data set cover?**

     *West\_Bounding\_Coordinate:* -180.000000

     *East\_Bounding\_Coordinate:* 180.000000

     *North\_Bounding\_Coordinate:* 83.623596

     *South\_Bounding\_Coordinate:* -90.000000

     - **What does it look like?**

       - **Does the data set describe conditions during a particular time period?**

         *Currentness\_Reference:*: publication date: 1996, 1998, Winter 1993/1994, 20000101; ground condition: 1994

         - **What is the general form of this data set?**

           *Geospatial\_Data\_Presentation\_Form:* vector digital data

           - **How does the data set represent geographic features?**

             1. **How are geographic features stored in the data set?**

                This is a Vector data set.
                It contains the following vector data types (SDTS terminology):
                - G-polygon (252)

                - **What coordinate system is used to represent geographic features?**

                  Horizontal positions are specified in geographic coordinates, that is, latitude and longitude.
                  Latitudes are given to the nearest 0.000001.
                  Longitudes are given to the nearest 0.000001.
                  Latitude and longitude values are specified in Decimal degrees.

                  The horizontal datum used is D\_WGS\_1984.  
                  The ellipsoid used is WGS\_1984.  
                  The semi-major axis of the ellipsoid used is 6378137.000000.  
                  The flattening of the ellipsoid used is 1/298.257224.

             - **How does the data set describe geographic features?**

               **cntry00**: The polygons represent the boundaries for the countries of the world, as they existed in 2000. (Source: ESRI) **FID**: Internal feature number. (Source: ESRI) *Sequential unique whole numbers that are automatically generated.* **Shape**: Feature geometry. (Source: ESRI) *Coordinates defining the features.* **FIPS\_CNTRY**: The FIPS code (two-letter) for the country. (Source: Department of Commerce, National Institute of Standards and Technology) | Formal codeset | | | --- | --- | | Codeset Name: | Federal Information Processing Standards Publication 10-4 | | Codeset Source: | National Institute of Standards and Technology | **GMI\_CNTRY**: The country code (three-letter) for the country from Global Mapping International. (Source: Global Mapping International) | Formal codeset | | | --- | --- | | Codeset Name: | Global Mapping International Codes | | Codeset Source: | Global Mapping International | **ISO\_2DIGIT**: The country code (two-letter) for the country from the International Organization for Standardization. (Source: International Organization for Standardization) | Formal codeset | | | --- | --- | | Codeset Name: | ISO 3166-1 Alpha-2 codes | | Codeset Source: | International Organization for Standardization | **ISO\_3DIGIT**: The country code (three-letter) for the country from the International Organization for Standardization. (Source: International Organization for Standardization) | Formal codeset | | | --- | --- | | Codeset Name: | ISO 3166-1 Alpha-3 codes | | Codeset Source: | International Organization for Standardization | **CNTRY\_NAME**: The country name. (Source: ArcWorld Supplement, Geographic and Global Issues, CIA Factbook) *Names for the features.* **LONG\_NAME**: The official country name. (Source: ArcWorld Supplement, Geographic and Global Issues, CIA Factbook) *Names for the features.* **SOVEREIGN**: The name of the sovereign for the country. (Source: ArcWorld Supplement, CIA Factbook) *Names for the features.* **POP\_CNTRY**: The 1994 estimated population of the country. (Source: National Center for Geographic Information and Analysis) | Value | Definition | | --- | --- | | -99999 | No population data available. | **CURR\_TYPE**: The type of currency for the country. (Source: ArcWorld Supplement) *Names for the features.* **CURR\_CODE**: The abbreviation (three-letter) for the currency of the country. (Source: ArcWorld Supplement) *Names for the features.* **LANDLOCKED**: Indicates if the country is landlocked. (Source: ArcWorld Supplement) | Value | Definition | | --- | --- | | Y | The country is landlocked. | | N | The country is not landlocked. | **SQKM**: The country area in square kilometers using an equal area projection. (Source: ESRI) *Calculated areas for the features.* **SQMI**: The country area in square miles using an equal area projection. (Source: ESRI) *Calculated areas for the features.* **COLOR\_MAP**: The number allows the country to be shaded unique from its neighbors. (Source: ESRI) | Range of values | | | --- | --- | | Minimum: | 1 | | Maximum: | 8 |

---


### Who produced the data set?

1. **Who are the originators of the data set?** (may include formal authors, digital compilers, and editors)

   - ESRI

   - **Who also contributed to the data set?**

     - **To whom should users address questions about the data?**

       > ESRI  
       > 380 New York Street  
       > Redlands, California 92373-8100  
       > USA  
       >
       > 909-793-2853 (voice)  
       > 909-793-5953 (FAX)  
       > info@esri.com

---


### Why was the data set created?

> For use with the book "Mapping Our World: GIS Lessons for Educators"

---


### How was the data set created?

1. **From what previous works were the data drawn?**

   **ESRI Data & Maps 2000** (source 1 of 4): > ESRI, 1996, ESRI Data & Maps 2000:, Environmental Systems Research Institute, Inc. (ESRI), Redlands, California, USA. *Type\_of\_Source\_Media:* CD�ROM *Source\_Contribution:* Attribute and geospatial data **Geographic and Global Issues** (source 2 of 4): > United States Department of Sta, Bureau of Intelligence and Research, Winter 1993/1994, Geographic and Global Issues Quarterly: Geographic and Global Issues Quarterly Volume 3, Number 4, United States Department of State, Bureau of Intelligence and Research, Washington, DC, USA. *Type\_of\_Source\_Media:* paper *Source\_Contribution:* Attribute data **CIA Factbook** (source 3 of 4): > United States Central Intelligence Agency, 20000101, The World Factbook 2000: The World Factbook 2000, United States Central Intelligence Agency, Washington, DC, USA. *Type\_of\_Source\_Media:* online *Source\_Contribution:* Attribute data **NCGIA** (source 4 of 4): > National Center for Geographic Information and Analysis, 199504, World Demography Project: National Center for Geographic Information and Analysis at University of California, Santa Barbara, Santa Barbara, California, USA. *Type\_of\_Source\_Media:* paper *Source\_Contribution:* Attribute data

   - **How were the data generated, processed, and modified?**

     Date: 08-Jan-2001 (process 1 of 1): Data sources used in this process: - ArcWorld Supplement- Geographic and Global Issues- CIA Factbook- World Demography Project

---


### How reliable are the data; what problems remain in the data set?

1. **How well have the observations been checked?**

   - **How accurate are the geographic locations?**

     The geospatial part of this data set was originally extracted from the ArcWorld Supplement database and then generalized (tolerance unknown). The positional accuracy is unknown.

     - **How accurate are the heights or depths?**

       - **Where are the gaps in the data? What is missing?**

         - **How consistent are the relationships among the observations, including topology?**

---


### How can someone get a copy of the data set?

> **Are there legal restrictions on access or use of the data?**
>
> > *Access\_Constraints:* Access granted to Licensee only.
> >
> > *Use\_Constraints:*: The data are provided by multiple, third party data vendors under license to ESRI for inclusion in the book "Mapping Our World: GIS Lessons for Educators." Licensee may use the data and related materials in quantities sufficient to meet Licensee's own internal needs, to be used solely in conjunction with the exercises and context of this book. The redistribution rights for this data set: Redistribution rights are granted by the data vendor for hard-copy renditions or static, electronic map images (e.g. .gif, .jpeg, etc.) that are plotted, printed, or publicly displayed with proper metadata and source/copyright attribution to the respective data vendor(s). Proprietary Rights and Copyright: Licensee acknowledges that the Data and Related Materials contain proprietary and confidential property of ESRI and its licensor(s). The Data and Related Materials are owned by ESRI and its licensor(s) and are protected by United States copyright laws and applicable international copyright treaties and/or conventions.

1. **Who distributes the data set?** (Distributor 1 of 1)

   > ESRI  
   > 380 New York Street  
   > Redlands, California 92373-8100  
   > USA  
   >
   > 800-447-9778 (voice)  
   > info@esri.com

   - **What's the catalog number I need to order this data set?**

     Included with the book "Mapping Our World: GIS Lessons for Educators"

     - **What legal disclaimers am I supposed to read?**

       > See use constraints.

       - **How can I download or order the data?**

         - **Availability in digital form:**

           |  |  |
           | --- | --- |
           | Data format: | SHP Size: 3.822 |
           | Media you can order: | ESRI Data & Maps 2000 CD�ROM Set is available only as part of ESRI® software. (Density 650 MB (megabytes)) (format ISO 9660) |

           - **Cost to order the data:**

         - **What hardware or software do I need in order to use the data set?**

           > To use this data requires software that supports ArcView® GIS shapefiles.

---


### Who wrote the metadata?

Dates:: Last modified: 19-Dec-2001 Metadata author:: > ESRI > 380 New York Street > Redlands, California 92373-8100 > USA > > 909-793-2853 (voice) > 909-793-5953 (FAX) > info@esri.com Metadata standard:: FGDC Content Standards for Digital Geospatial Metadata (FGDC-STD-001-1998) Metadata extensions used:: - <http://www.esri.com/metadata/esriprof80.html>

---

Generated by mp version 2.7.3 on Thu Jan 03 09:57:38 2002
