# Supplementary material for: Modelling the timing of migration of a partial migrant bird using ringing and observation data: a case study with the Song Thrush in Italy
Source: Mov Ecol. 2023 Aug 1;11:47. doi: 10.1186/s40462-023-00407-z (PMC10391980; doi:10.1186/s40462-023-00407-z)

Figure S3.1: a), b), and c) Dates when the estimated number of encounters starts deviating from that expected from the capture/observations of stationary individuals; they were calculated on the whole (a) or the rarefied (b) ringing datasets, or the eBird dataset (c). Isolines represent areas where the migration date occurs at the same time. Months are divided into ten-day periods (‘decades’ *sensu* the key concepts document of the EU Birds Directive; e.g. Jan 1, Jan 2, Jan 3). Isolines labels should be interpreted as the first day of the corresponding decade, e.g. isoline JAN1 should be read as "01 January", JAN2 as "11 January ", JAN3 as "21 January " and so on. d), e) and f) Sensitivity analyses associated with panels a), b) and c), respectively. Isolines include areas with the same sensitivity value (in days) of the estimated date of onset of the pre-nuptial migration.


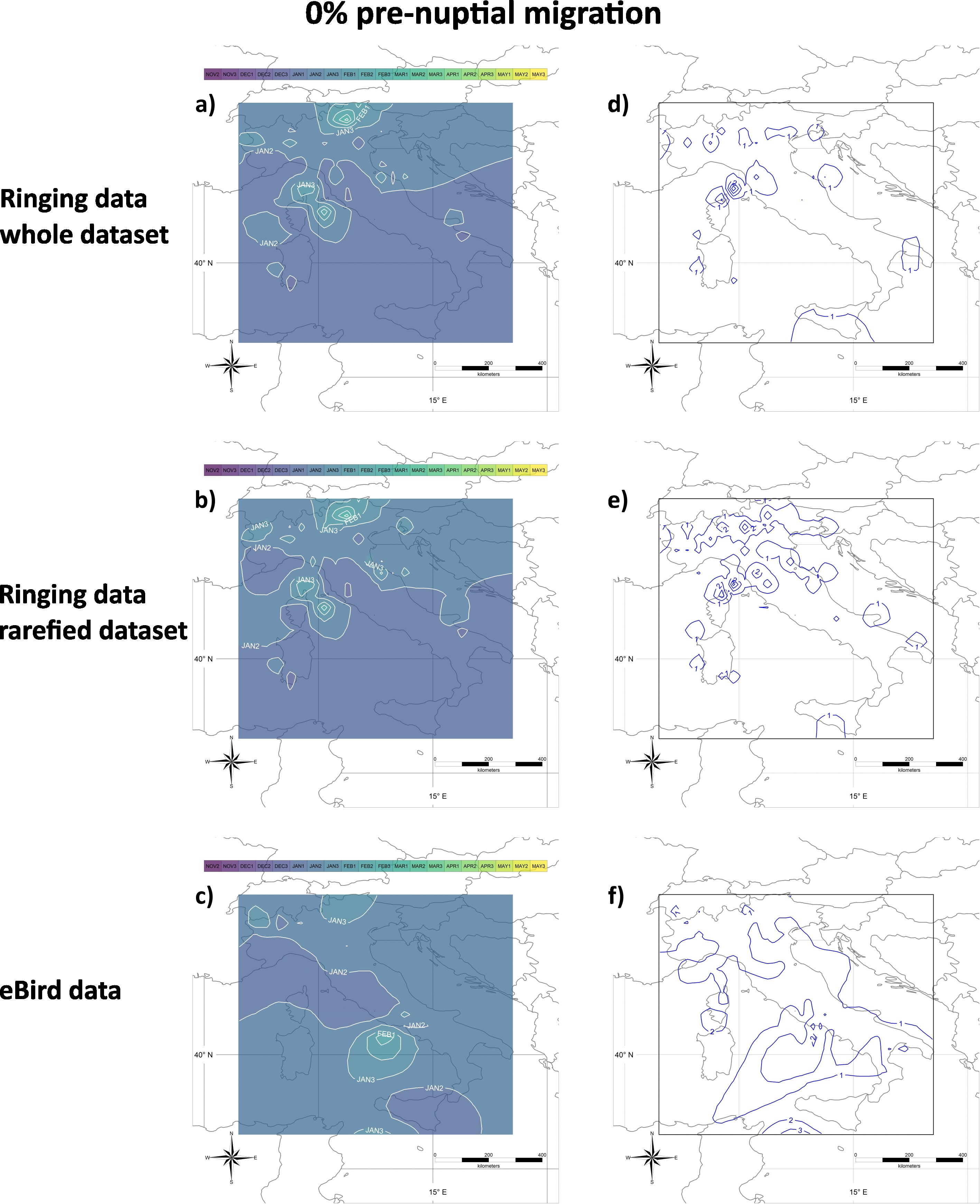


Figure S3.2: a), b), and c) Dates when 1% more encounters than expected from the capture/observation of stationary individuals do occur; they were calculated on the whole (a), or the rarefied (b) ringing datasets or the eBird dataset (c). Isolines represent areas where the migration date occurs at the same time. Months are divided into ten-day periods (‘decades’ *sensu* the key concepts document of the EU Birds Directive; e.g. Jan 1, Jan 2, Jan 3). Isolines labels should be interpreted as the first day of the corresponding decade, e.g. isoline JAN1 should be read as "01 January", JAN2 as "11 January ", JAN3 as "21 January " and so on. d), e) and f) Sensitivity analyses associated with panels a), b), and c), respectively. Isolines incorporate areas with the same sensitivity value (in days) of the estimated date of onset of the pre-nuptial migration.


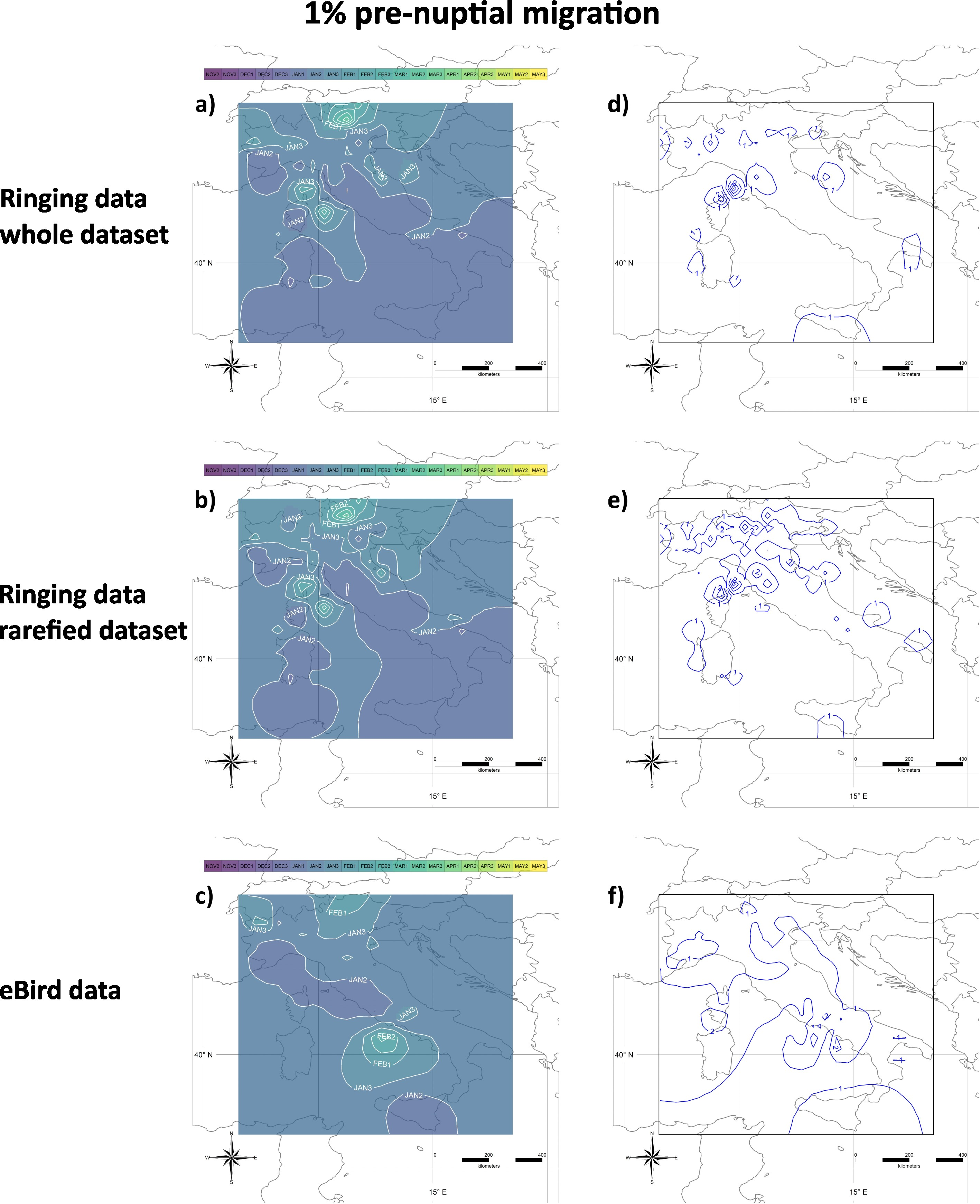


Figure S3.3: a), b) and c) Dates indicate when 10% more encounters than expected from the capture/observation of stationary individuals do occur; they were calculated on the whole (a) or the rarefied (b) ringing datasets, or the eBird dataset (c). Isolines represent areas where the migration date occurs at the same time. Months are divided into ten-day periods (‘decades’ *sensu* the key concepts document of the EU Birds Directive; e.g. Jan 1, Jan 2, Jan 3). Isolines labels should be interpreted as the first day of the corresponding decade, e.g. isoline JAN1 should be read as "01 January", JAN2 as "11 January ", JAN3 as "21 January " and so on. d), e) and f) Sensitivity analyses associated with panels a), b), and c), respectively. Isolines incorporate areas with the same sesitivity values (in days) of the estimated date of onset of the pre-nuptial migration.


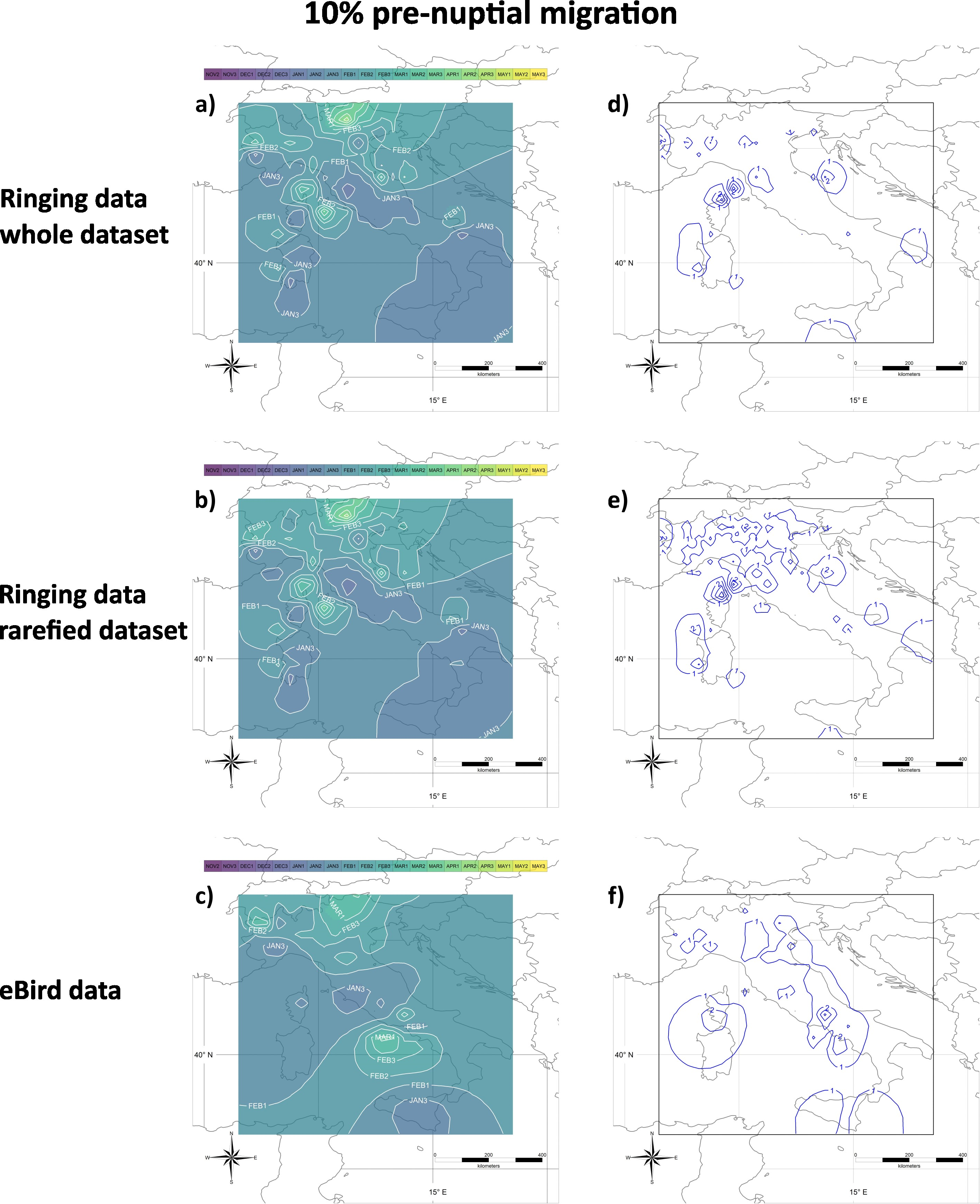

Supplement: Supplementary file 3 — Supplementary Material 3 [file 40462_2023_407_MOESM3_ESM.docx]
