# Supplementary material for: Modelling the timing of migration of a partial migrant bird using ringing and observation data: a case study with the Song Thrush in Italy
Source: Mov Ecol. 2023 Aug 1;11:47. doi: 10.1186/s40462-023-00407-z (PMC10391980; doi:10.1186/s40462-023-00407-z)

Figure S4.1: a), b), and c) Dates when the estimated number of encounters starts deviating from that expected from the capture/observation of stationary individuals; they were calculated on the whole (a) or the rarefied (b) ringing datasets, or the eBird dataset (c). Isolines represent areas where the migration date occurs at the same time. Months are divided into ten-day periods (‘decades’ *sensu* the key concepts document of the EU Birds Directive; e.g. Oct 1, Oct 2, Oct 3). Isolines labels should be interpreted as the first day of the corresponding decade, e.g. isoline OCT1 should be read as "01 October", OCT2 as "11 October", OCT3 as "21 October" and so on. d), e) and f) Sensitivity analyses associated with panels )a, b) and c) respectively. Isolines include areas with the same sensitivity value (in days) of the estimated median date of the post-nuptial migration.


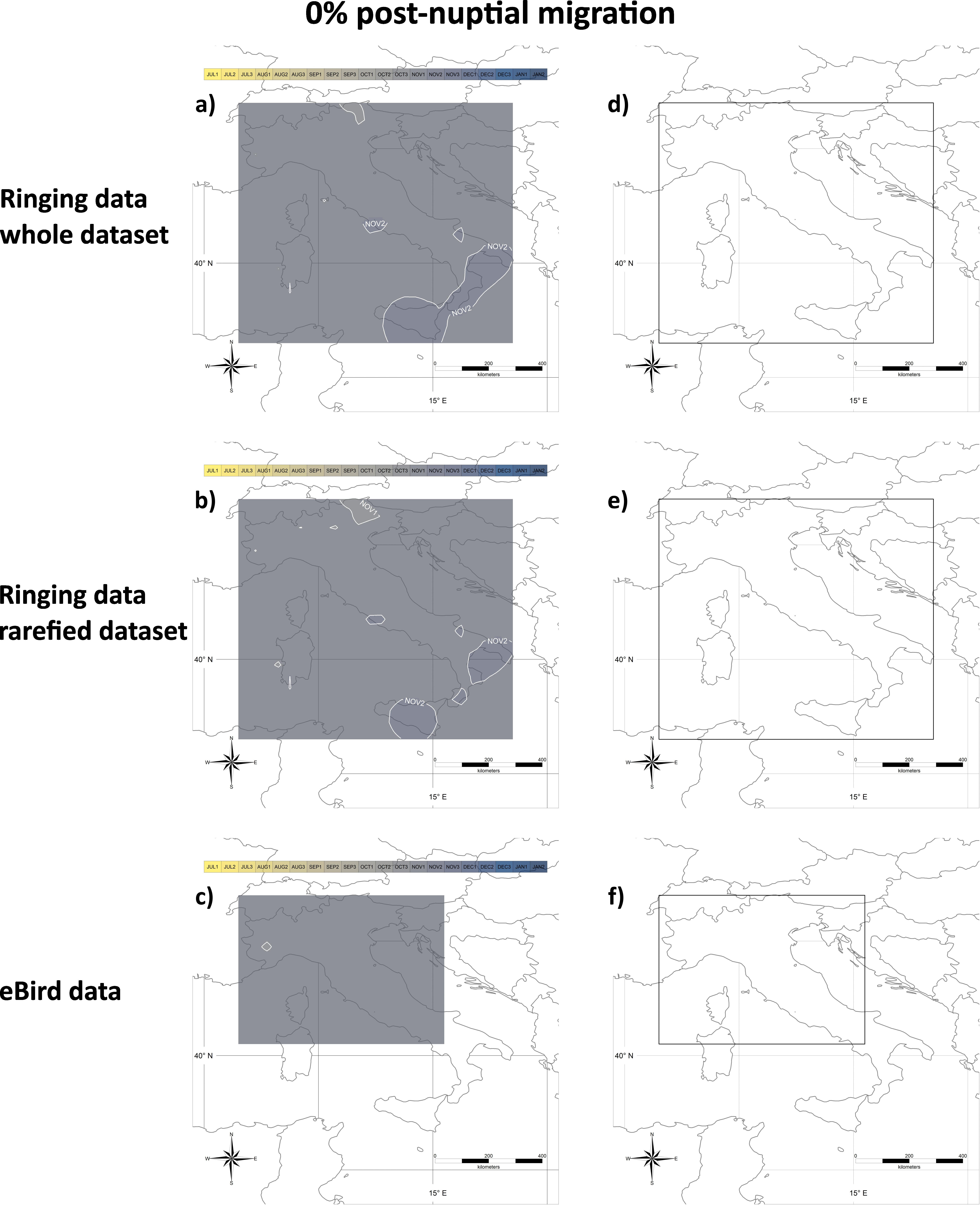


Figure S4.2: a), b), and c) Dates when 1% more encounters than expected from the capture/observation of stationary individuals do occur; they were calculated on the whole (a) or the rarefied (b) ringing datasets, or the eBird dataset (c). Isolines represent areas where the migration date occurs at the same time. Months are divided into ten-day periods (‘decades’ *sensu* the key concepts document of the EU Birds Directive; e.g. Oct 1, Oct 2, Oct 3). Isolines labels should be interpreted as the first day of the corresponding decade, e.g. isoline OCT1 should be read as "01 October", OCT2 as "11 October", OCT3 as "21 October" and so on. d), e) and f) Sensitivity analyses associated with panels a), b) and c) respectively. Isolines include areas with the same sensitivity value (in days) of the estimated median date of the post-nuptial migration.
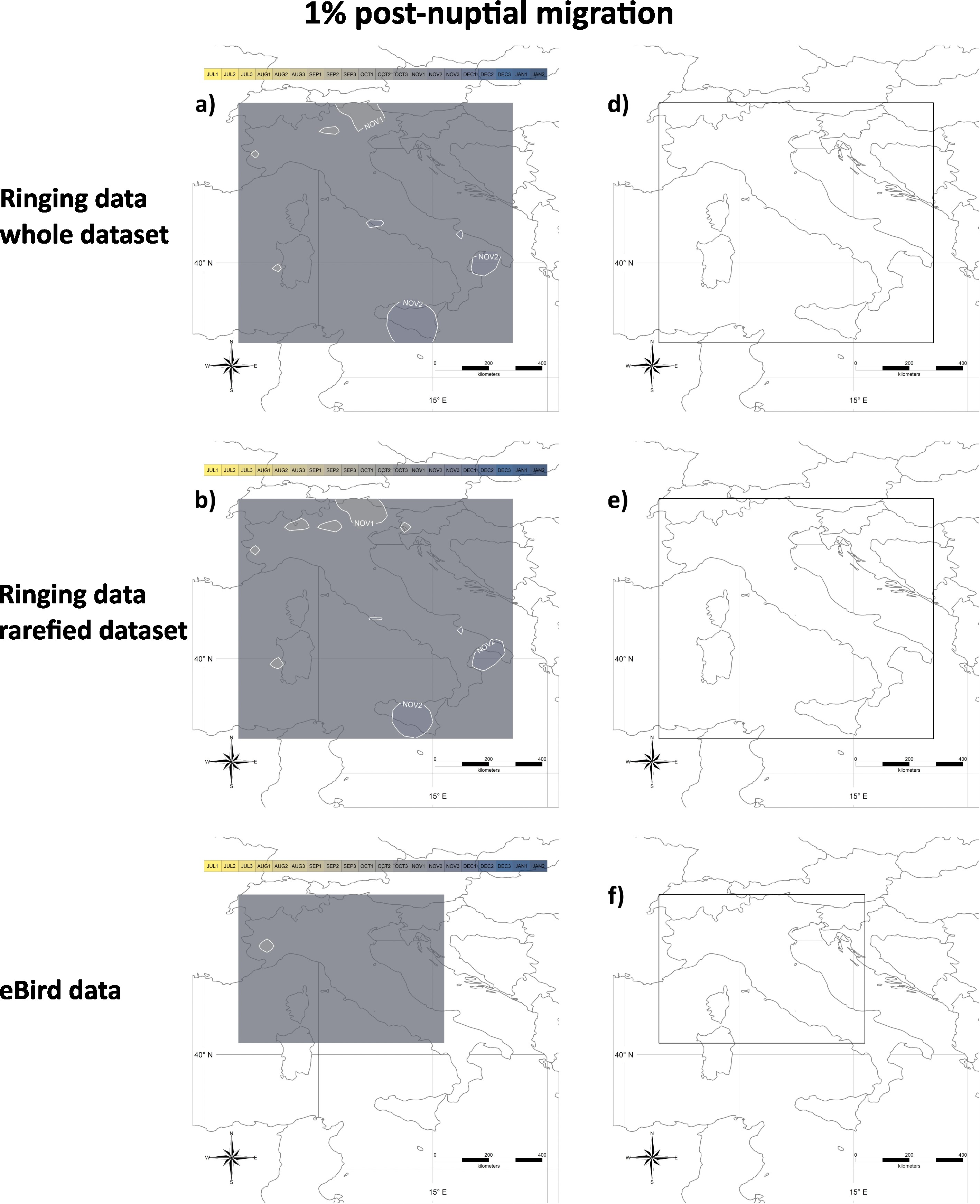


Figure S4.3: a), b), and c) Dates when 10% more encounters than expected from the capture/observation of stationary individuals do occur; they were calculated on the whole (a) or the rarefied (b) ringing datasets, or the eBird dataset (c). Isolines represent areas where the migration date occurs at the same time. Months are divided into ten-day periods (‘decades’ *sensu* the key concepts document of the EU Birds Directive; e.g. Oct 1, Oct 2, Oct 3). Isolines labels should be interpreted as the first day of the corresponding decade, e.g. isoline OCT1 should be read as "01 October", OCT2 as "11 October", OCT3 as "21 October" and so on. d), e) and f) Sensitivity analyses associated with panels a), b) and c) respectively. Isolines include areas with the same sensitivity value (in days) of the estimated median date of the post-nuptial migration.


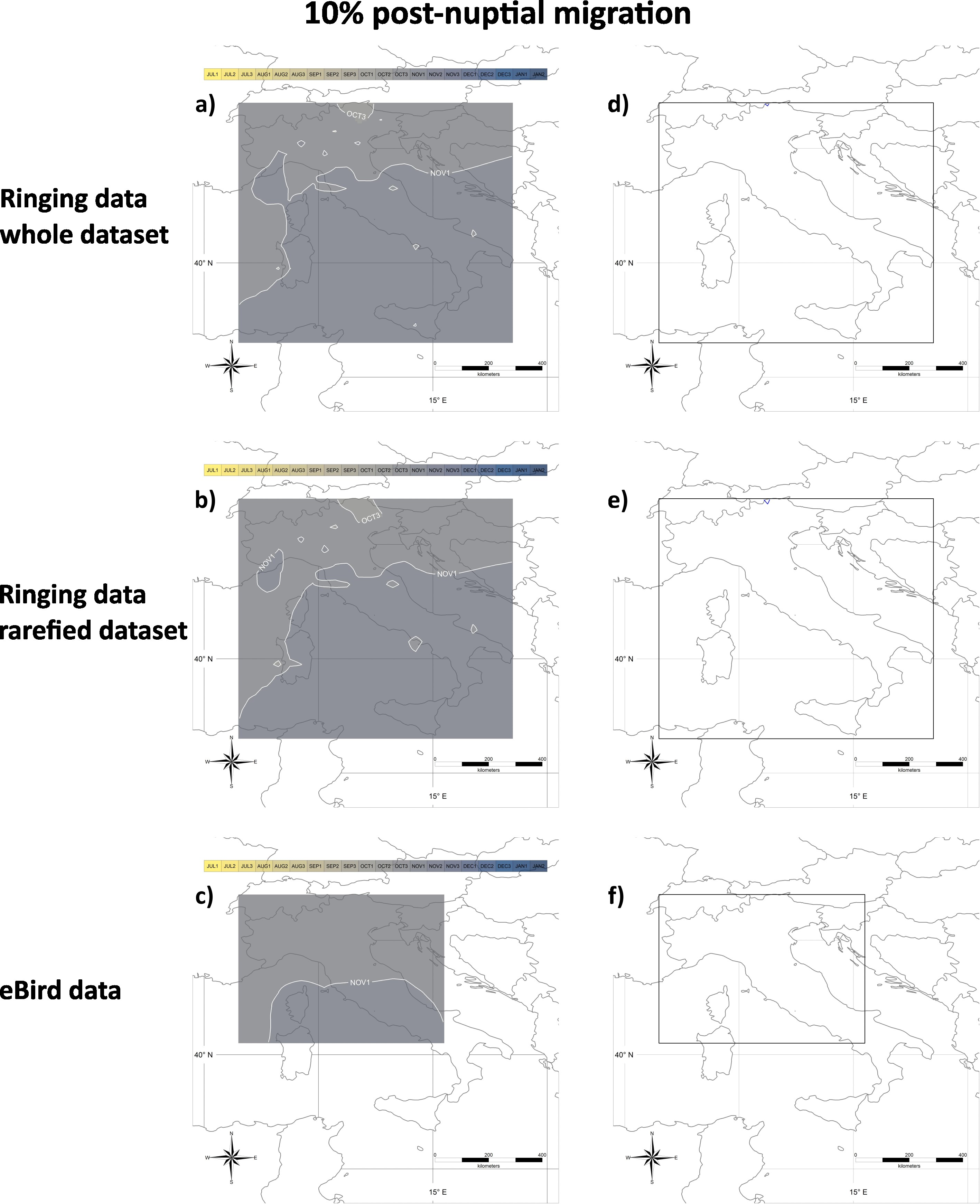

Supplement: Supplementary file 4 — Supplementary Material 4 [file 40462_2023_407_MOESM4_ESM.docx]
